# Editorial: Semantics and natural language processing in agriculture

**DOI:** 10.3389/frai.2025.1718114

**Published:** 2025-11-25

**Authors:** Mathieu Roche, Brett M. Drury

**Affiliations:** 1 CIRAD, UMR TETIS, Montpellier, France; 2 TETIS, Univ. de Montpellier, AgroParisTech, CIRAD, INRAE, Montpellier, France; 3 Liverpool Hope University, Hope Park, Liverpool, United Kingdom

**Keywords:** semantics, nlp, text mining, ontology, lexical resources

## 1 Introduction

The application of data science in agriculture enables the analysis of diverse datasets using methods such as machine learning, deep learning, computer vision, text mining (Drury and Roche, 2019), and large language models (LLMs) (Li et al., 2025; Shaikh et al., 2024). These techniques support tasks such as crop yield prediction and the early detection of plant and animal diseases by integrating heterogeneous data sources, including sensor readings, textual reports, satellite imagery, and plant images (Jabed et al., 2024). For practitioners and decision makers, data-driven insights provide a robust evidence base for promoting more efficient and sustainable agricultural practices (Rozenstein et al., 2024).

Within digital agriculture, the management of textual data and semantic information remains a major challenge. Semantics assigns unique identifiers to concepts, reducing ambiguity and enabling data integration across the agricultural value chain. This enhances interoperability and strengthens agricultural information systems. In this context, *Agrisemantics* has emerged as a dedicated field focusing on the application of semantic technologies in agriculture (Drury et al., 2019).

The urgency of this research is underscored by global challenges such as rising food prices, diminishing arable land due to climate change, and growing population pressures. This Research Topic brings together four contributions, including original research, brief reports, and data studies, each illustrating recent advances in semantics and text mining for agricultural applications.

## 2 Semantics and text mining studies

The Research Topic is organised into four principal themes: Semantics for Agri-food Systems, Semantic Analysis for Food Safety, Text Mining for Plant Health, and Lexicon Construction for Organic Residue Valorization. Each theme is represented by a single contribution.

### 2.1 Semantics for agri-food systems

Chaib et al. proposed a systematic methodology for developing agricultural ontologies by combining the Godet and MyChoice approaches. Drawing on stakeholder interviews and extending to complementary construction techniques, their study demonstrates the value of integrating multiple methodologies to enhance ontology design in agriculture.

### 2.2 Semantic analysis or food safety

Food safety remains a global concern, exemplified by incidents such as *Operao Carne Fraca* in Brazil and the 2008 Chinese Milk Scandal. However, even uncontaminated food products can generate perceptions of risk among consumers. Aline et al. investigated subjective consumer beliefs regarding the risks of infant food through a cross-national study. Their findings revealed intra-cultural variations in risk perception, providing insights that may inform governmental communication strategies to mitigate public concern.

### 2.3 Text mining for plant health

Addressing data sparsity in agricultural text classification, Jiang et al. utilised unlabelled data and Generative Adversarial Networks to fine-tune a pre-trained language model (PLM). Their approach achieved improved performance compared with traditional methods across multiple tasks. By reducing dependence on costly labelled datasets, this method lowers barriers to adopting text mining in agricultural research.

### 2.4 Lexicon construction for organic residue valorization

Rakotomalala et al. created a domain-specific lexicon to support research on organic residue valorization in developing countries. Combining expert knowledge with NLP techniques, the authors identified 2,079 relevant terms, which are publicly available (https://doi.org/10.18167/DVN1/HNZZSI). The lexicon has since enabled semantically driven analyses of a large corpus compiled from multiple scientific databases and repositories.

## 3 Conclusion

Text mining and semantic approaches are playing an increasingly significant role in agricultural research. Since the launch of this Research Topic, the contributions have collectively attracted more than 11,000 views. These studies also lay the groundwork for future applications of LLMs in addressing agricultural challenges (De et al., 2025; Li et al., 2025). The continued support of organizations such as GODAN (Global Open Data for Agriculture and Nutrition) and the FAO will be essential for advancing research that integrates semantics and text mining at the forefront of agricultural innovation and practice.
